# The Impact of Post-graduate Year and Program Accreditation Status on In-Training Examination Performance in Orthopaedic Surgery

**DOI:** 10.7759/cureus.39053

**Published:** 2023-05-15

**Authors:** Jason Silvestre, John D Kelly, Robert H Wilson, Charles L Nelson

**Affiliations:** 1 Orthopaedic Surgery, Howard University College of Medicine, Washington, USA; 2 Orthopaedic Surgery, Perelman School of Medicine, Philadelphia, USA; 3 Orthopaedics, Howard University Hospital, Washington, USA; 4 Orthopaedics, Perelman School of Medicine, Philadelphia, USA

**Keywords:** acgme, in-training, examination, orthopaedic, surgery, residency

## Abstract

Introduction

The progression of medical knowledge competency during surgical residency training is poorly understood. This study measures the acquisition of medical knowledge as orthopedic surgery residents advance during training and the impact of accreditation status on orthopedic in-training examination (OITE) performance.

Methods

Orthopedic surgery residents taking the OITE during 2020 and 2021 were included. Residents were grouped into cohorts by post-graduate year (PGY) and Accreditation Council for Graduate Medical Education (ACGME) accreditation status. Comparisons were made with parametric tests.

Results

Eight thousand eight hundred and seventy-one ACGME-accredited residents (89%) and 1,057 non-ACGME-accredited residents (11%) were evenly distributed by the PGY level (range, 19-21%). Residents in both ACGME- and non-ACGME-accredited residency programs had a significant increase in OITE performance at each PGY level (P<0.001). At ACGME-accredited programs, OITE performance increased from PGY1 (51%), PGY2 (59%), PGY3 (65%), PGY4 (68%), and PGY5 (70%) (P<0.001). There were progressively smaller percentage increases in OITE performance during accredited residency training (range, 2-8%), but this increase was linear in non-accredited residency training (range, 4%). At each PGY level, residents at accredited programs outperformed their counterparts at non-accredited programs (P<0.001).

Conclusion

OITE performance increases during residency training. Among ACGME-accredited residents, performance on the OITE progresses rapidly during junior years and plateaus during senior years. Residents in ACGME-accredited residency programs outperform their counterparts in non-accredited residency programs. More research is needed to understand optimal training environments that promote medical knowledge acquisition during orthopedic surgery residency.

## Introduction

The Accreditation Council for Graduate Medical Education (ACGME) outlines core competency training for surgical residency training in the United States (US). Medical knowledge is an ACGME-defined core competency for independent clinical practice [1-3]. The ACGME serves to provide accreditation standards for postgraduate medical training in the US and, increasingly, internationally [4]. The World Health Organization has claimed that standardized and accredited global medical training is a strategic priority for the twenty-first century [5]. Currently, however, there is a paucity of research outlining differences in medical knowledge competency during orthopedic surgery residency [6-7].

The wide breadth of medical knowledge required during orthopedic surgery residency training is tested annually in the orthopedic in-training examination (OITE) [8]. The American Academy of Orthopaedic Surgeons (AAOS) administers this examination to residents in orthopedic surgery allowing them to gauge their mastery of medical knowledge against their peers. Insights into the progression of medical knowledge during residency can help accrediting bodies in surgical education understand the correlation between medical knowledge acquisition and post-graduate year (PGY) during residency training. Furthermore, elucidation of an objective benchmark for OITE performance can help surgical residency programs identify residents who are not progressing relative to their peers.

In this study, we sought to determine the degree to which OITE performance increases during orthopedic surgery residency training. We hypothesized that OITE performance would increase during each successive PGY level but would increase in smaller rates at senior (PGY4-5) relative to junior (PGY1-3) resident levels. Furthermore, we hypothesized that orthopedic surgery residents in ACGME-accredited programs would outperform their international counterparts in non-ACGME-accredited programs.

## Materials and methods

The AAOS provided OITE scores from the 2020-2021 and 2021-2022 academic years. OITE performance data included all orthopedic surgery residents at ACGME-accredited residency programs in the United States and select international residents at non-ACGME-accredited residency programs. These international residents were included if their respective residency program voluntarily opted into the OITE administration. Due to the de-identified nature of all data, this study received review exemption status based on the policies of the Institutional Review Board at Howard University.

Each year, the OITE is designed to consist of 275 multiple choice questions that cover 10 clinical content domains (i.e., basic sciences, foot and ankle, hand and wrist, oncology, pediatrics, shoulder and elbow, spine, sports, trauma) covering established treatment principles and modalities in orthopedic surgery. A few practice management questions are occasionally included. The exam is assembled into two sections where residents are allotted 210 minutes for each section for a total of seven hours. The OITE Examination Committee of the AAOS vets questions through a peer-reviewed process, and the final set is ratified by the Board of Directors. All final questions are intended for scoring, but questions are occasionally flagged for poor test performance (e.g., confusing language, inaccurate). In 2020, eight questions were removed from final scoring (n=267 scored questions). In 2021, twelve questions were removed from final scoring (n=263 scored questions). The AAOS scores the OITE based on the raw number of questions answered correctly and reports the examinee's raw score along with percentile rank among the national resident class at a specific post-graduate year level.

OITE performance was defined in two ways: the number and percentage of OITE questions answered correctly. Parametric and chi square tests were used to compare OITE performance by PGY level within accredited and non-accredited residency programs. This tested the hypothesis that OITE performance increases during residency training. Percentage increases in OITE performance based on annual promotion were compared between junior resident (PGY1-3) and senior resident (PGY4-5) levels. A final separate analysis was conducted to compare the OITE performance between residents at accredited and non-accredited programs. This tested the hypothesis that ACGME accreditation status is associated with greater OITE performance.

All statistical tests were performed using GraphPad Prism 9 software (GraphPad Software, Inc., La Jolla, CA). OITE performance data were analyzed with D’Agostino-Pearson Omnibus normality tests and presented as means with standard deviations. Relative proportions of tested content between the 2020 and 2021 OITEs were compared with chi-square tests. Statistical tests were two-tailed and considered significant if P<0.05.

## Results

OITE performance data from 9,928 unique administrations were included in this study (Table 1). There were more residents in ACGME-accredited training programs (89%) than non-accredited programs (11%) with even distribution at the PGY1 (n=1,924, 19%), PGY2 (n=2,048, 21%), PGY3 (n=2,030, 20%), PGY4 (n=1,996, 20%), and PGY5 (n=1,930, 19%) levels. Tested content domains were similar between the 2020 and 2021 OITEs (P>0.05, Table 2). Trauma (13%), pediatrics (12%), hip and knee (12%), and basic sciences (11%) were the most commonly tested domains.

**Table 1:** Orthopedic Surgery Residents Taking the Orthopedic In-Training Examination PGY, post-graduate year; ACGME, Accreditation Council for Graduate Medical Education

Year	Total Number of Orthopedic Surgery Residents (% of Total)											
	PGY 1		PGY 2		PGY 3		PGY 4		PGY 5		Total	
	ACGME	Non-ACGME	ACGME	Non-ACGME	ACGME	Non-ACGME	ACGME	Non-ACGME	ACGME	Non-ACGME	ACGME	Non-ACGME
2020	860 (90)	99 (10)	908 (89)	108 (11)	900 (88)	119 (12)	877 (89)	104 (11)	860 (89)	107 (11)	4,405 (89)	537 (11)
2021	879 (91)	86 (9)	911 (88)	121 (12)	900 (89)	111 (11)	895 (88)	120 (12)	881 (91)	82 (9)	4,466 (90)	520 (10)
Total	1,739 (90)	185 (10)	1,819 (89)	229 (11)	1,800 (89)	230 (11)	1,772 (89)	224 (11)	1,741 (90)	189 (10)	8,871 (89)	1,057 (11)

**Table 2:** Distribution of Tested Content on Consecutive Orthopedic In-Training Examinations Chi square tests demonstrated no significant differences in tested content domains between the 2020 and 2021 OITE administrations OITE, orthopedic in-training examination

Content Domain	Proportion of Scored OITE, %		P
	2020	2021	
Basic Sciences	11	11	1.0
Foot and Ankle	9	10	0.815
Hand and Wrist	7	10	0.613
Hip and Knee	11	14	0.531
Oncology	9	9	1.0
Pediatrics	10	13	0.517
Practice Management	1	0	1.00
Shoulder and Elbow	8	10	0.633
Spine	12	8	0.359
Sports	7	5	0.570
Trauma	15	12	0.544
Total	100	100	--

**Figure 1:**
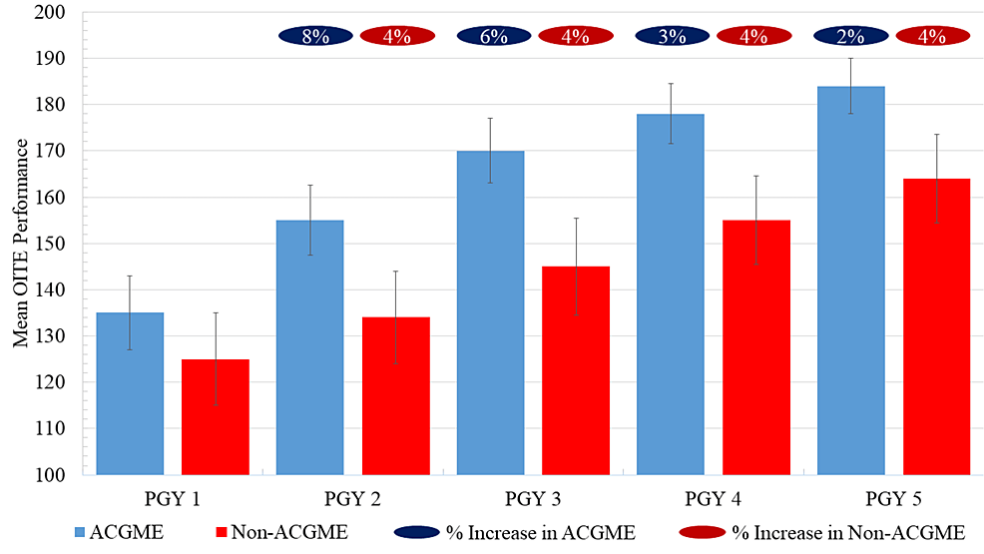
Comparison of Orthopedic In-Training Examination Performance by Accreditation Status and Post-Graduate Year of Training OITE performance defined by the mean number of questions answered correctly; error bars represent standard deviation; Student's t tests demonstrate higher OITE scores for residents in ACGME accredited programs at each PGY level (P<0.05) PGY, post-graduate year; ACGME, Accreditation Council for Graduate Medical Education; OITE, orthopedic in-training examination

There was a significant increase in mean OITE performance at each PGY level for residents at both ACGME- and non-ACGME accredited residency programs (P<0.001, Figure 1). There were progressively smaller percentage increases in OITE performance across PGY levels during ACGME-accredited residency training: PGY1-2 (8% increase, n=20 questions), PGY2-3 (6% increase, n=15 questions), PGY3-4 (3% increase, n=8 questions), and PGY4-5 (2% increase, n=6 questions). In non-ACGME-accredited residency training, there was a linear increase in OITE performance: PGY1-2 (4% increase, n=9 questions), PGY2-3 (4% increase, n=11 questions), PGY3-4 (4% increase, n=10 questions), and PGY4-5 (4% increase, n=10 questions).

Residents at accredited programs outperformed their counterparts at non-accredited programs at each training level: PGY1 (51% vs 47%, P<0.001), PGY2 (59% vs 51%, P<0.001), PGY3 (65% vs 55%, P<0.001), PGY4 (68% vs 59%, P<0.001), and PGY5 (70% vs 62%, P<0.001) (Table 3). This disparity was smallest at the PGY1 level (4% difference) and greatest at the PGY3 level (10% difference).

**Table 3:** Comparison of Orthopedic In-Training Examination Performance by Accreditation Status PGY, post-graduate year; ACGME, Accreditation Council for Graduate Medical Education

Accreditation Status	Proportion of OITE Answered Correctly, %									
	PGY 1	P	PGY 2	P	PGY 3	P	PGY 4	P	PGY 5	P
ACGME	51	<0.001	59	<0.001	65	<0.001	68	<0.001	70	<0.001
Non-ACGME	47		51		55		59		63	

## Discussion

Graduates of ACGME-accredited orthopedic surgery residency programs must pass the American Board of Orthopaedic Surgeons (ABOS) Part I written examination. Residency programs maintain accreditation, in part, by their ability to successfully graduate residents that pass Part I on the first attempt. To date, OITE performance is the best barometer for future ABOS Part I written examination performance [9-17]. OITE performance benchmarks from this study can be used to monitor knowledge acquisition during residency, which can be helpful to identify underperforming residents. Ultimately, we confirmed our hypothesis that OITE performance increases to a smaller degree at senior resident levels, but this only occurred among accredited programs. In contrast, the increase in OITE performance among non-accredited residents was linear. Reasons for this remain unknown but likely stem from different resident training factors and a testing threshold effect, which makes it harder to score higher as performance increases. Lastly, residents in accredited programs outperformed their international counterparts in non-accredited programs, which was likely due to the emphasis on the ABOS Part I written examination in the US and its correlation with OITE performance [9-17].

The ACGME has shifted toward competency-based training, which requires objective assessment across six core competencies needed for independent practice [1-3]. Medical knowledge is routinely evaluated by in-training examination performance, which standardizes evaluation to a national standard. In this study, we demonstrated that PGY is correlated with increases in OITE performance during residency, which varies by accreditation status. However, the clinical significance of this progression in OITE performance on resident competency is outstanding. That is, whether ten or twenty additional multiple-choice questions answered correctly translates into greater competency remains unknown. Of note, previous linking studies performed by the AAOS have demonstrated that a minimum score of 169 OITE questions (63% correct) corresponds to the minimum passing score on the ABOS Part I Written Examination [18,19]. Several other studies have also demonstrated that poor OITE performance correlates with failure on the ABOS Part I Written Examination [9-16]. In the future, OITE performance can provide additional data points to inform orthopedic surgery milestones in an objective manner [20]. Ideally, this would span all orthopedic sub-specialties, which would be a powerful instrument for competency-based resident assessment in the US.

The standardization of global medical training is a strategic priority for the World Health Organization [5]. Historically, the ACGME has provided the gold standard for accreditation of US postgraduate medical education but has recently achieved international reach [4]. The benefits of ACGME accreditation include the provision of specific standards regarding program faculty, facilities, curriculum structure, and leadership [21]. Ultimately, uniform standards can translate into better training environments for surgical residents. In this study, accreditation status was associated with higher OITE performance, but the potential for confounding factors should be highlighted. For example, international residents may be less incentivized to perform well on the OITE relative to their American counterparts [17]. Additionally, the structure of medical education differs between the US and other countries, which may lead to unaccounted-for differences in residency training [22,23]. Unfortunately, we could not assess the specific international programs that voluntarily opted into OITE administration. Many of these residency programs were Canadian given their proximity to the US, but this could not be quantified. Conversely, the primary strength of this study was its universal capture of all accredited residents in the US where previous research has included only a sub-set of programs [9-16].

There were several limitations to this study. First, the clinical relevance of additional questions answered correctly on the OITE remains unknown. Future studies should correlate the acquisition of medical knowledge with clinical competency to see if translates into better objective outcomes. Evidence-based recommendations are also needed to inform orthopedic surgery milestones across additional dimensions of medical knowledge [10]. Importantly, there is evidence to suggest a lack of correlation between standardized exam scores and clinical performance [11]. Second, this study is a snapshot of OITE performance from 2020 and 2021. Thus, insights regarding OITE performance by training level and accreditation status will naturally evolve over time. Increasingly, the nature and purpose of the OITE have shifted to mirror the ABOS Part I Board Examination. As such, disparities in OITE performance may change with time. Finally, aggregate reporting of OITE performance data limited additional analyses by resident characteristics like race and gender as well as examination characteristics like sub-specialty area.

## Conclusions

In summary, OITE performance increases during orthopedic surgery residency training. Residents in ACGME-accredited programs outperform their international colleagues in non-ACGME-accredited programs. Ultimately, this study provides an objective benchmark that measures how residency promotion affects medical knowledge acquisition during orthopedic surgery residency. More research is needed to understand optimal training environments that promote medical knowledge acquisition during orthopedic surgery residency training, including the potential benefits of ACGME accreditation.
